# Improving the efficiency of a CIGS solar cell to above 31% with Sb_2_S_3_ as a new BSF: a numerical simulation approach by SCAPS-1D

**DOI:** 10.1039/d3ra07893k

**Published:** 2024-01-08

**Authors:** Md. Ferdous Rahman, Mithun Chowdhury, Latha Marasamy, Mustafa K. A. Mohammed, Md. Dulal Haque, Sheikh Rashel Al Ahmed, Ahmad Irfan, Aijaz Rasool Chaudhry, Souraya Goumri-Said

**Affiliations:** a Advanced Energy Materials and Solar Cell Research Laboratory, Department of Electrical and Electronic Engineering, Begum Rokeya University Rangpur 5400 Bangladesh ferdousapee@gmail.com; b Facultad de Química, Materiales-Energía, Universidad Autónoma de Querétaro (UAQ) Santiago de Querétaro Querétaro C.P. 76010 Mexico; c College of Remote Sensing and Geophysics, Al-Karkh University of Science Al-Karkh Side, Haifa St. Hamada Palace Baghdad 10011 Iraq; d Department of Electronics and Communication Engineering, Hajee Mohammad Danesh Science and Technology University Dinajpur 5200 Bangladesh; e Department of Electrical, Electronic and Communication Engineering, Pabna University of Science and Technology Pabna 6600 Bangladesh; f Department of Chemistry, College of Science, King Khalid University P.O. Box 9004 Abha 61413 Saudi Arabia; g Department of Physics, College of Science, University of Bisha P.O. Box 551 Bisha 61922 Saudi Arabia; h Physics Department, Colleges of Science and General Studies, Alfaisal University P.O. Box 50927 Riyadh 11533 Saudi Arabia sosaid@alfaisal.edu

## Abstract

The remarkable performance of copper indium gallium selenide (CIGS)-based double heterojunction (DH) photovoltaic cells is presented in this work. To increase all photovoltaic performance parameters, in this investigation, a novel solar cell structure (FTO/SnS_2_/CIGS/Sb_2_S_3_/Ni) is explored by utilizing the SCAPS-1D simulation software. Thicknesses of the buffer, absorber and back surface field (BSF) layers, acceptor density, defect density, capacitance–voltage (*C*–*V*), interface defect density, rates of generation and recombination, operating temperature, current density, and quantum efficiency have been investigated for the proposed solar devices with and without BSF. The presence of the BSF layer significantly influences the device's performance parameters including short-circuit current (*J*_sc_), open-circuit voltage (*V*_oc_), fill factor (FF), and power conversion efficiency (PCE). After optimization, the simulation results of a conventional CIGS cell (FTO/SnS_2_/CIGS/Ni) have shown a PCE of 22.14% with *V*_oc_ of 0.91 V, *J*_sc_ of 28.21 mA cm^−2^, and FF of 86.31. Conversely, the PCE is improved to 31.15% with *V*_oc_ of 1.08 V, *J*_sc_ of 33.75 mA cm^−2^, and FF of 88.50 by introducing the Sb_2_S_3_ BSF in the structure of FTO/SnS_2_/CIGS/Sb_2_S_3_/Ni. These findings of the proposed CIGS-based double heterojunction (DH) solar cells offer an innovative method for realization of high-efficiency solar cells that are more promising than the previously reported traditional designs.

## Abstract 2

The remarkable performance of copper indium gallium selenide (CIGS)-based double heterojunction (DH) photovoltaic cells is presented in this work.

## Graphical abstract

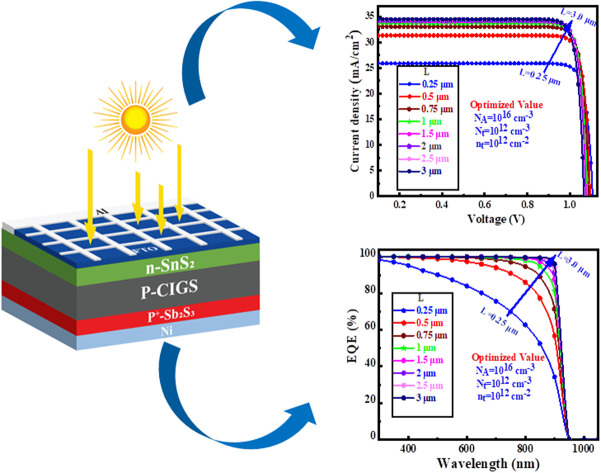

## 1 Introduction

One of the main objectives of scientists and researchers across the globe is the development of renewable and eco-friendly energy sources to reduce the detrimental effects of CO_2_ emissions generated by the usage of fossil fuels. Renewable sources of energy, such as photovoltaic (PV) cells, are crucial in meeting the increasing energy demand and promoting green energy.^1–4^ The PV power has been growing at an approximate rate of 8.3% annually.^5^ Solar energy has become an important source for the replacement of fossil fuel since it is abundant, eco-friendly, and renewable.^6^ A device called a PV cell is utilized to transform solar energy into electrical power, which is a plentiful, efficient, and affordable source of electricity. There are two types of PV cells: thin and bulk. These cells harness solar radiation through photoconductivity to generate electrical energy. Thin film solar cells (TFSCs) are becoming increasingly popular as they offer a cost-effective alternative to traditional solar cells, resulting in their widespread adoption in various applications. Dye-sensitized solar cells (DSSCs) have been investigated extensively as TFSCs that transform light into electrical energy for the past twenty years.^7^ Despite their potential, these solar cells are also notorious for their instability.^8^ The CIGS TFSCs are currently in high demand because of their impressive cost-effectiveness and exceptional PCE. As a result, they have become an increasingly sought-after renewable energy source within the global community. The effectiveness of CIGS in outdoor conditions is exceptional, showcasing its high efficiency in converting solar power. This makes CIGS-based TFSCs a preferred option in renewable energy.^9^ The chalcogenide material utilized as the absorber layer with p-type characteristics in the CIGS solar cells is the best-suited option, which greatly enhances its overall efficiency.^10^ Despite its relatively high manufacturing cost, CIGS stands out as a top performer among thin-film technologies, surpassing even hydrogenated amorphous silicon (a-Si:H) and cadmium telluride (CdTe) solar cells with impressive performance capabilities.^11^ Incorporating rare and costly materials, such as gallium and indium, significantly increases the production cost of CIGS solar cells. Thus, to mitigate this challenge, the CIGS layer thickness is needed to minimized for optimizing cost-effectiveness.^12^ The CIGS is composed of a combination of four different elements. The CIGS absorber has an absorption coefficient of 10^5^ cm^−1^ and a energy gap (*E*_g_) of 1.1 eV.^13,14^ The energy difference between the CIGS absorber layer's conduction and valence bands can be adjusted by varying the ratio of gallium and indium, resulting in a bandgap that can range from 1.02 eV to 1.69 eV.^15^ According to the report of First Solar, the maximum power conversion efficiency for CdTe TFSCs is found experimentally to be 22.1%. On the other hand, the greatest theoretical efficiencies in the range from 28% to 30% of the CdTe solar cell with absorber bandgap of 1.5 eV is determined.^16^ The maximum conversion efficiency for silicon solar cells is obtained to be 27.1%, which is attained by Trina Solar. Conversely, currently, the c-Si solar cells have a record efficiency of 26.7%, compared to an intrinsic limit of approximately 29%.^17^ A fascinating concept in the solar energy is the Shockley–Queisser limit, which demonstrates a theoretical maximum efficiency of a single p–n junction solar cell reaching 30%. With a promising potential, the CIGS-based PV cells strive to surmount the Shockley–Queisser limit as their efficiency nears its threshold. The CIGS TFSCs have demonstrated remarkable efficiency with realizable and promising option for large-scale commercial implementation. These solar cells have also demonstrated outstanding stability at high temperatures, making them an ideal choice for aerospace applications, where minimizing weight and volume are critical considerations.^18^ The CIGS solar cells have undoubtedly progressed, yet challenges persist, primarily centered on efficiency and cost considerations. On the efficiency front, while there have been notable improvements, the CIGS cells still lag behind the traditional Si-based counterparts, with flexible CIGS cells achieving approximately 20.3% efficiency on rigid glass substrates. Addressing absorption limitations, despite CIGS's advantageous direct bandgap and higher absorption coefficient than Si, there remains an inability to effectively capture all sunlight photons. Researchers have explored strategies such as incorporating additional absorber layers like CuInSe_2_ beneath the CIGS layer to enhance absorption. Moreover, advancements in the ZnO layer, particularly the integration of magnesium (Zn_1−*x*_Mg_*x*_O), have demonstrated improved performance by redirecting high-energy photons into the main absorber layer. Turning to the cost aspect, while CIGS cells leverage abundant materials, concerns arise from the cost of indium and gallium, crucial elements for achieving high efficiency. The manufacturing process, involving intricate layers (ZnO:Al/ZnO/CdS/CIGS/MO), adds complexity and cost. Further, the reliance on vacuum-based deposition techniques (such as sputtering or evaporation) for CIGS layers introduces additional expenses compared to alternative thin-film technologies. Navigating these efficiency and cost challenges is paramount for advancing the practical viability of CIGS solar cells.^19–22^

In scientific literature, it has been reported that the thicknessess of CIGS absorber layer ranged from from 1 to 4 μm are employed in the CIGS-based TFSCs. Moreover, empirical evidence suggests that the efficiency of CIGS-based TFSCs tends to fall below 28% and 24% in theoretical and experimental settings, respectively.^10,13,23–25^ In pursuing more affordable and efficient solar cells, a groundbreaking idea has emerged with Sb_2_S_3_ semiconducting material as a back surface field (BSF) for the CIGS-based PV cells. By reducing the thickness and cost of the CIGS absorber layer, this innovative approach promises to make solar energy more accessible and sustainable. With a energy gap varying from 1.60 to 1.62 eV and exceptional stability, the Sb_2_S_3_ has emerged as a popular choice in TFSC technology. A noteworthy aspect of Sb_2_S_3_ is that it comprises the elements Sb and S, which are abundantly available on Earth. Hence, incorporating a thick layer of Sb_2_S_3_ in industrial CIGS solar cells can effectively reduce their production costs.^25^ The Sb_2_S_3_ stands out as the quintessential candidate for an absorber material in solar cells, given its proven experimental and theoretical competencies. This study shows that the Sb_2_S_3_ is the optimal compound for the BSF layer in the CIGS PV cells in comparison with other BSF-assisted structures.^25^ In the initial simulation process, the CdS buffer layer is replaced with SnS_2_ layers to make it cadmium-free, resulting in a new and innovative FTO/SnS_2_/CIGS/Sb_2_S_3_ heterojunction solar cell design. The optimization of buffer layer thicknesses followed this modification. The CIGS layer has been optimized through a meticulous variation of doping concentration, defect density, thickness, and interface defect density using diverse buffer layers. The adjustment of the thickness and doping concentration of the Sb_2_S_3_ BSF layer has also been executed for optimization. After exploring multiple options for back and front electrodes, we have successfully optimized our selection to include nickel (Ni) and aluminum (Al) for their superior performance in our proposed solar cells. After a comprehensive examination of the solar cell structure, an optimal configuration has been obtained, bringing the study to a satisfactory conclusion. Our revolutionary CIGS-based PV cells, with SnS_2_ buffers and Sb_2_S_3_ BSF layer, have shattered all previous records with remarkable PCEs of 31.15%. These extraordinary results have set a new standard in the field of solar cells, outperforming all CIGS-based solar cells reported so far.^9,13,26–28^

The investigation of electrical performance in the proposed CIGS-based PV cells involved the integration of the Sb_2_S_3_ BSF layer. Key parameters—open-circuit voltage (*V*_oc_), fill factor (FF), short-circuit current density (*J*_sc_) and efficiency (*η*)—are examined with a focus on optimizing doping concentration, defect density, thickness, and interface defect density. The arrangement of the paper is as follows: in Section 2, the simulated structure and its corresponding parameters are presented. Section 3 elaborates on the mathematical modeling, while Section 4 showcases the outcomes and subsequent discussions. Section 5 provides a relative investigation between the proposed work and recent publications. Lastly, Section 6 concludes the paper.

## 2 Device construction and simulation methodology

**Fig. 1:**
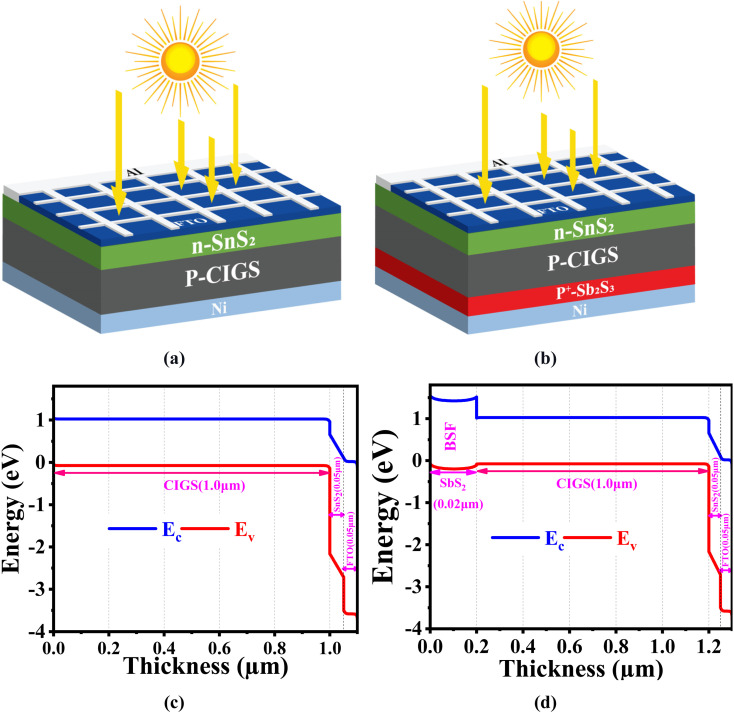
The schematic structural design of suggested CIGS-based PV cell (a) without BSF, (b) with BSF, and the energy band diagram (c) without BSF, and (d) with BSF.

The Solar Cell Capacitance Simulator (SCAPS) application in one dimension has been employed for simulation purposes to analyze the recently constructed PV cell with the structure FTO/SnS_2_/CIGS/Sb_2_S_3_/Ni. The SCAPS-1D, is a promising program created by the Department of Electronic and Information Systems at the University of Ghent, Belgium. The PV cell architectures' optoelectronic properties can be analyzed and predicted using essential equations including continuity and electrostatic potential equations in a steady-state environment.^29^ In Fig. 1(a), we can observe the conventional configuration (Ni/Sb_2_S_3_/CIGS/SnS_2_/FTO/Al) of a CIGS solar cell. The p-type CIGS absorber with an energy gap (*E*_g_) of 1.1 eV^13^ forms a junction with an n-type SnS_2_ buffer layer having an *E*_g_ of 2.24 eV.^30^ The energy gap of the FTO window layer is 3.6 eV.^31^ The proposed CIGS configuration (Ni/Sb_2_S_3_/CIGS/SnS_2_/FTO/Al) with a 0.2 μm Sb_2_S_3_ BSF layer is illustrated in Fig. 1(b), providing a significant reduction in material cost while maintaining performance. The energy band representation of our proposed solar cell, showcasing the bandgap and thickness of each layer, is brilliantly exhibited in Fig. 1(c), derived from SCAPS simulation-generated data on energy band panels. The band bending between the junctions of Sb_2_S_3_ and CIGS, which can be observed in Fig. 1(d), is influenced by the different levels of doping concentrations employed in this study. The Sb_2_S_3_ layer is used as BSF in our proposed structure (FTO/SnS_2_/CIGS/Sb_2_S_3_/Ni). A BSF plays a vital role in reducing carrier recombination at the back surface of the heterojunction TFSC, thus improving the efficiency of solar cells. The insertion of the Sb_2_S_3_ as a BSF layer between the absorber and the rear electrode will create sufficient built-in potential (Fig. 1(d)), thereby limiting the flow of minority electrons towards the back surface by reflecting back to front electrode. In addition, the valence band maximum of the BSF layer should be well-aligned with the valence band maximum of the absorber material. This alignment reduces the energy barrier for holes to move from the CIGS absorber layer to back contact through the Sb_2_S_3_ BSF. This proper energy level alignment helps to ensure that holes can easily move across the interface without losing energy and reducing the probability of recombination (Fig. 1(d)). The proper band alignment at CIGS/Sb_2_S_3_ interface would be effective to diminish the chances of electrons and holes recombining within the proposed heterojunction device, consequently improving the overall collection efficiency.^32,33^

In PV devices like solar cells, the work function is essential for determining the efficiency of electron transfer and collection. The alignment of work functions at different interfaces within the device affects the generation and extraction of electric charges, impacting overall device performance. In our proposed structure we used nickel (Ni) as a back contact layer. Although some researchers also used Au and Mo as a back contact layer with Sb_2_S_3_ layer.^34–38^ This innovative solar cell design consists of a p-type CIGS absorber layer with a thickness of 1.0 μm, a p^+^ type Sb_2_S_3_ BSF layer of 0.2 μm on the back Ni layer, an FTO window layer measuring 0.05 μm and an n-type SnS_2_ buffer layer of 0.05 μm. Aluminum (Al) is the selected material for the front grid contact because of its high work function of 4.06 eV, allowing it to efficiently extract and transport the generated charge carriers in a solar cell.^11^ The bandgap, mobility, electron affinity, thermal speed, doping, the effective density of states, and a host of other characteristics can all be graded in the SCAPS. Various lighting spectra can be used for testing solar cells, such as AM0, AM1.5D, white, monochromatic and the standard test condition (STC) that corresponds to a global air mass of 1.5 (AM1.5G).^39^ The specifications for the layers utilized in solar cells are shown in Table 1, along with their parameters values.

**Table 1:** Layer properties used in Al/FTO/SnS_2_/CIGS/Sb_2_S_3_/Ni solar cell^30,31,39,48^^a^

Parameters (unit)	FTO	SnS_2_	CIGS	Sb_2_S_3_
Layer type	Window	ETL	Absorber	BSF
Conductivity type	n^+^	n	p	p^+^
Thickness (μm)	0.05	0.05	1.0*	0.2
Bandgap (eV)	3.6	2.24	1.1	1.62
Electron affinity (eV)	4	4.24	4.2	3.70
Dielectric permittivity (relative)	9	10	13.6	7.08
CB effective DOS (cm^−3^)	2.2 × 10^18^	2.2 × 10^18^	2.2 × 10^18^	2.0 × 10^19^
VB effective DOS (cm^−3^)	1.8 × 10^19^	1.8 × 10^19^	1.8 × 10^19^	1.0 × 10^19^
Electron thermal velocity (cm s^−1^)	1 × 10^7^	1 × 10^7^	1 × 10^7^	1 × 10^7^
Hole thermal velocity (cm s^−1^)	1 × 10^7^	1 × 10^7^	1 × 10^7^	1 × 10^7^
Electron mobility (cm^2^ V^−1^ s^−1^)	100	50	100	9.8
Hole mobility (cm^2^ V^−1^ s^−1^)	25	50	25	10
Donor density, *N*_D_ (cm^−3^)	1 × 10^18^	1 × 10^15^	0	0
Acceptor density, *N*_A_ (cm^−3^)	0	0	1 × 10^16^*	1 × 10^15^
Defect type	SA	SA	SD	SD
Defect density (cm^−3^)	1 × 10^12^	1 × 10^12^	1 × 10^12^	1 × 10^12^

a SA single acceptor, SD single donor, (*) variable field.

The band alignment significantly influences the flow of current through the heterojunction. Table 2 shown the interface parameter of the proposed structure. The use of numerical design approaches is crucial in analyzing device outputs and identifying highly efficient PV cells. In the scientific world of solar energy research, the one-dimensional SCAPS-1 simulator has been viewed as a viable tool for planning and evaluation of polycrystalline TFSCs.^29^ The SCAPS-1D software can simulate a solar device with up to seven different semiconductor layers. The heterojunction TFSCs are also quantitatively assessed using this numerical method, which makes use of a set of parameters for a variety of materials. Furthermore, by utilizing the entered similarity parameters of the used films in the simulator, it may also clarify gadget qualities evaluated by other scientists, leading to the model's attainment of reliability. Numerous studies investigating the perspective of finding heterojunction photovoltaic systems using numerical simulations by the SCAPS-1D program have been published.^40–45^ The PV performance metrics, including *V*_oc_, *J*_sc_, FF, efficiency, and spectrum responses, which are measured both theoretically and experimentally, are validated in their attempts. Previous research has revealed that the device outputs computed numerically match the experimental results exactly, confirming the validity of the SCAPS-1D simulator.^43–47^ Consequently, heterojunction PV structures can be effectively modeled and simulated using the dependable SCAPS-1D tool without sacrificing generality. The TFSC has been designed and simulated in this investigation using the SCAPS-1D simulation program. In order to obtain the greatest performance of the predicted PV device construction, all perceptions that are desirable have been taken into consideration when using the practically accessible material's characteristics as indicated in the previous investigations.

**Table 2:** Interface factors used in Al/FTO/SnS_2_/CIGS/Sb_2_S_3_/Ni solar cell

Parameters (unit)	Sb_2_S_3_/CIGS interface	CIGS/SnS_2_ interface
Defect type	Neutral	Neutral
Electron capture cross-section, *σ*_e_ (cm^2^)	1 × 10^19^	1 × 10^19^
Hole capture cross-section, *σ*_p_ (cm^2^)	1 × 10^19^	1 × 10^19^
Defect position above the highest *E*_V_ (eV)	0.06	0.06
Interface defect density (cm^−2^)	1 × 10^12^	1 × 10^12^

## 3 Mathematical modeling

The solar cell device output can be numerically modeled using the SCAPS-1D created by Burgelman *et al.*^29^ Under steady-state conditions, semiconductor compounds are governed by a one-dimensional equation. The relationship between charge density and the electric fields (*E*) at the p–n junction can be written using the symbols below,^49^1
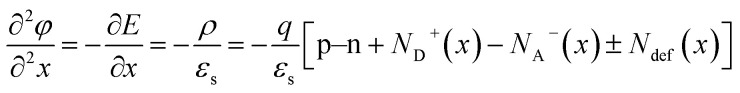


This equation involves some variables: electrostatic potential, charge, the medium's relative permittivity, electrons and the hole, and there are donor and acceptor densities and acceptor and donor's defect density.

While the PSC device's carrier continuity expression can be composed as follows^50^2
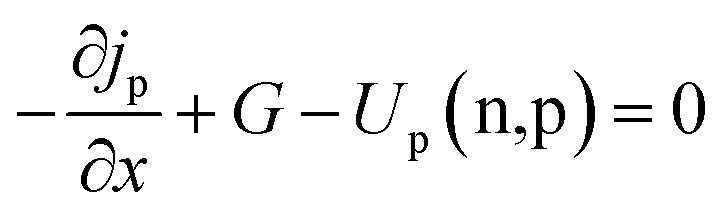
3
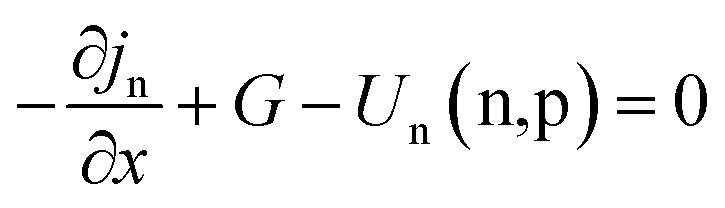
*G* is the carrier generation rate, and *U*_n_(n,p) is the electron and hole recombination rate, respectively. *j*_p_ and *j*_n_ are known as the hole and electron current densities.^51^

Additionally, the following equation can be used to get the carrier current density.^52^4
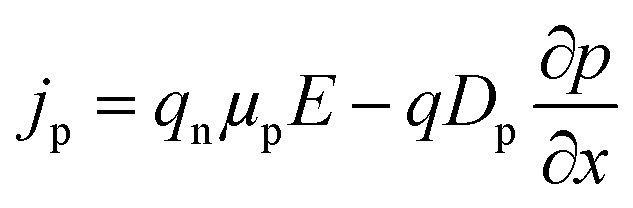
5
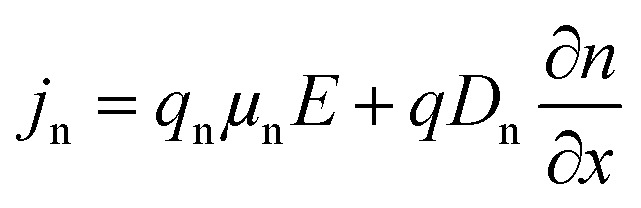
In this context, the charge is represented by the letter *q*, the carrier mobilities are represented by the letters *μ*_p_ and *μ*_n_, and the carrier diffusion coefficients are defined by the letter *D*_p_ and *D*_n_.^53^

It should be noted that the SCAPS-1D program extracts the fundamental equation for current density, recombination, and generation rate for solar cells.

## 4 Results and discussions

The full analysis and investigation of intrinsic solar cell characteristics are covered in the seven subsections that comprise the results and discussion segment numbered from 4.1 to 4.8.

### 4.1 Effects of absorber layer thickness and acceptor density on performances of CIGS solar cell

**Fig. 2:**
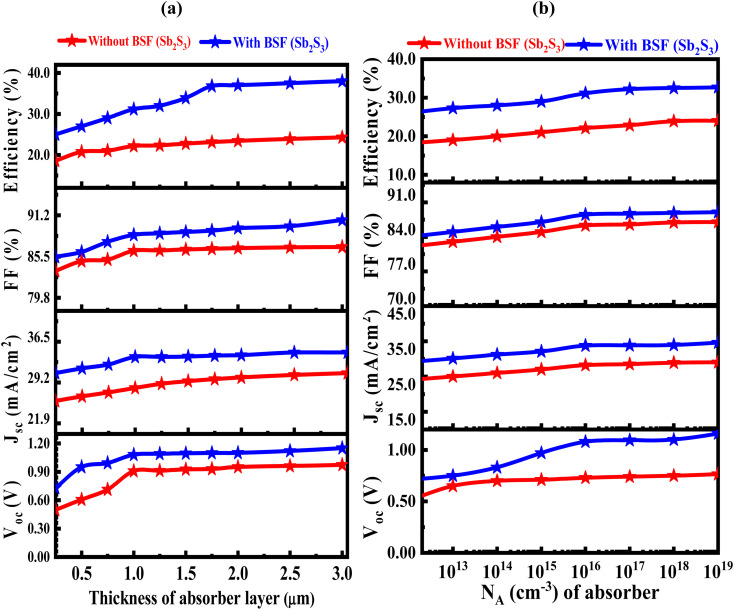
(a) The impact of changing the CIGS thickness layer, and (b) The impact of the acceptor density in the CIGS layer with and without Sb_2_S_3_ BSF layer.

The output characteristics parameters of CIGS-based PV cells are affected by the absorber's thickness, as shown in Fig. 2(a). As the CIGS absorber thickness rises, the *J*_sc_ exhibits a positive correlation and rises in the devices. It reaches a saturated value of 28.21 mA cm^−2^ without BSF and 33.75 mA cm^−2^ for the Sb_2_S_3_ BSF layer at 1.0 μm. Then, because of the recombination kinetics that affects the charge separation in the suggested SC device, the value of the *J*_sc_ keeps marginally rising on further increasing thickness. It is also projected that the net absorption will increase up to an absorber thickness of 1.0 μm, speeding the rate of extraction. Due to the extensive photon absorption, this starts a good generation of electrons in the active layer.^54^ The variation of *V*_oc_ concerning the absorber thickness varies between 0.25 μm and 3.0 μm, is also presented concurrently. It peaks at 1.0 μm thickness and then shows a steady trend due to the recombination kinetics that predominates at thicker layers.^55^ After detail investigate 0.91 and 1.08 V was found is the optimized value of *V*_oc_ without and with BSF, respectively. While the FF of the device shows a typical proportional increase with an increase in absorber thickness and is predicted to reach saturation at a thickness of 1.0 μm. In the absence of a BSF, the fill factor of the CIGS PV cell exhibits an increase from 83.52% to 85.83%, whereas the introduction of a thin Sb_2_S_3_ layer as the BSF leads to a further boost in fill factor from 85.46% to 89.23%. The efficiency of the SC reaches a maximum at a thickness of around 1.0 μm due to the direct effects of thickness's on the *J*_sc_, *V*_oc_, and FF. It gets a saturated PCE value of 22.14% without BSF and 31.15% for the Sb_2_S_3_ BSF layer. Due to a larger bandgap, the CIGS active layer may have an advantage since photons are absorbed in a broader range of wavelengths.^56^

To evaluate its implications on the functionality of the suggested SC device, *N*_A_ is altered between 1 × 10^12^ and 10^19^ cm^−3^ in the CIGS absorber layer. The influence of the *N*_A_ on the absorber (CIGS) layer has been observed over range 10 (ref. 12) to 10^19^ cm^−3^, as seen in Fig. 2(b). The goal was to increase the *V*_oc_ from 0.91 to 1.08 V and obtain a saturated *J*_sc_ value of 33.75 mA cm^−2^ by changing the *N*_A_ value in the CIGS material from 10^12^ to 10^19^. The FF remains constant until an *N*_A_ value of 10^16^, after which it starts to increase. At high doping concentrations, Auger recombination becomes more prominent. An electron and a hole can recombine to release energy known as auger recombination and this releases energy that is transferred to either an electron or a hole instead of being radiatively emitted as light. This non-radiative recombination increases with carrier concentration, leading to higher current losses. On the other hand, at very high doping levels, carrier mobility may be reduced. This reduction in mobility can lead to decreased carrier transport efficiency, resulting in higher resistivity and increased current loss.^57,58^ Eventually, it reaches a maximum value of 88.50% at an *N*_A_ value of 10^16^. When the *N*_A_ value exceeds 10^19^ cm^−3^, excessive carrier concentration causes recombination and increases scattering, which ultimately enhances the recombination rate of the electron–holes, therefore, it represents insignificant impact on the *N*_A_ density.^54^

### 4.2 Impact of absorber layer's carrier concentration on G–R profile of CIGS solar cell

**Fig. 3:**
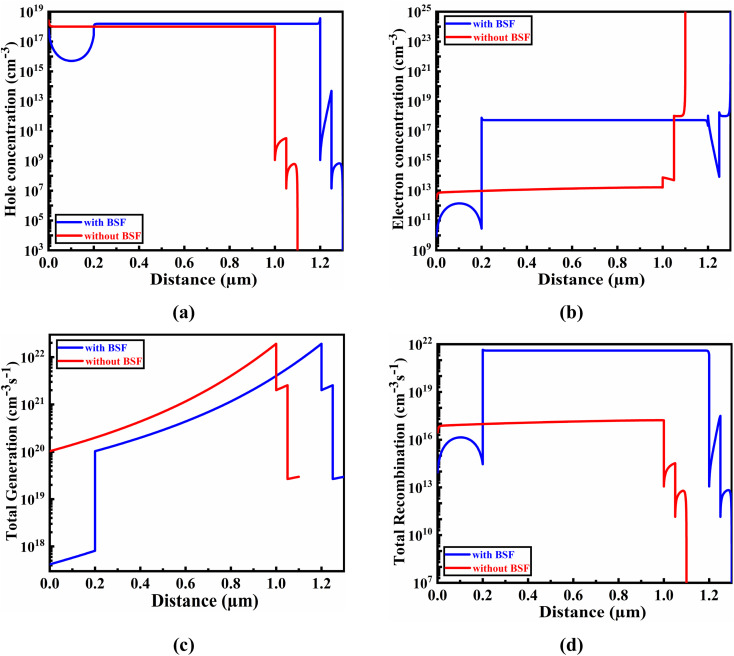
Impact of (a) hole, and (b) electron carrier concentrations, as well as overall (c) total generation, and (d) total recombination, in relation to the thickness of the absorber layer.

The correlation between electron and hole carrier concentration and the G–R profile, concerning the CIGS absorber layer's thickness, is depicted in Fig. 3. This simulated results provide insight into the effect of the Sb_2_S_3_ BSF layer on the relationship between these factors.

The acceptor concentration variation in the absorber is responsible for the slightly higher hole concentration in CIGS with BSF (Sb_2_S_3_) compared to that without BSF. This, in turn, leads to a more efficient DOS in the valence bands.^31^ In contrast, the CIGS absorber with Sb_2_S_3_ BSF has a greater electron concentration compared to the CIGS absorber without BSF. A comprehensive investigation of the carrier creation and recombination behaviors of CIGS-based PV cells with and without BSF has been conducted to reveal their potentiality, in comparison to materials utilized as absorbers include previously reported organic, inorganic, and compound semiconductors. The carrier concentration and defect density are kept constant at specific and adjusted values for the comparison.^59–62^ Therefore, we may conclude that using BSF layer in CIGS-based heterojunctions offers great promise as extremely effective materials employed as absorbers in TFSCs. They enhance carrier generation while reducing electron–hole recombination, leading to an overall improvement in performance. The presence of BSF layer contribute to create electric field interface of Sb_2_S_3_/CIGS which in turn enhances the electron concentration as well as the generation rate of carriers as verified in Fig. 3(b) and (c), respectively. The excessive electron–hole pair density greater than 10^17^ cm^−3^ also increases the recombination.

### 4.3 Impact of performance due to concurrent changing of CIGS absorber layer's thickness and defects density

**Fig. 4:**
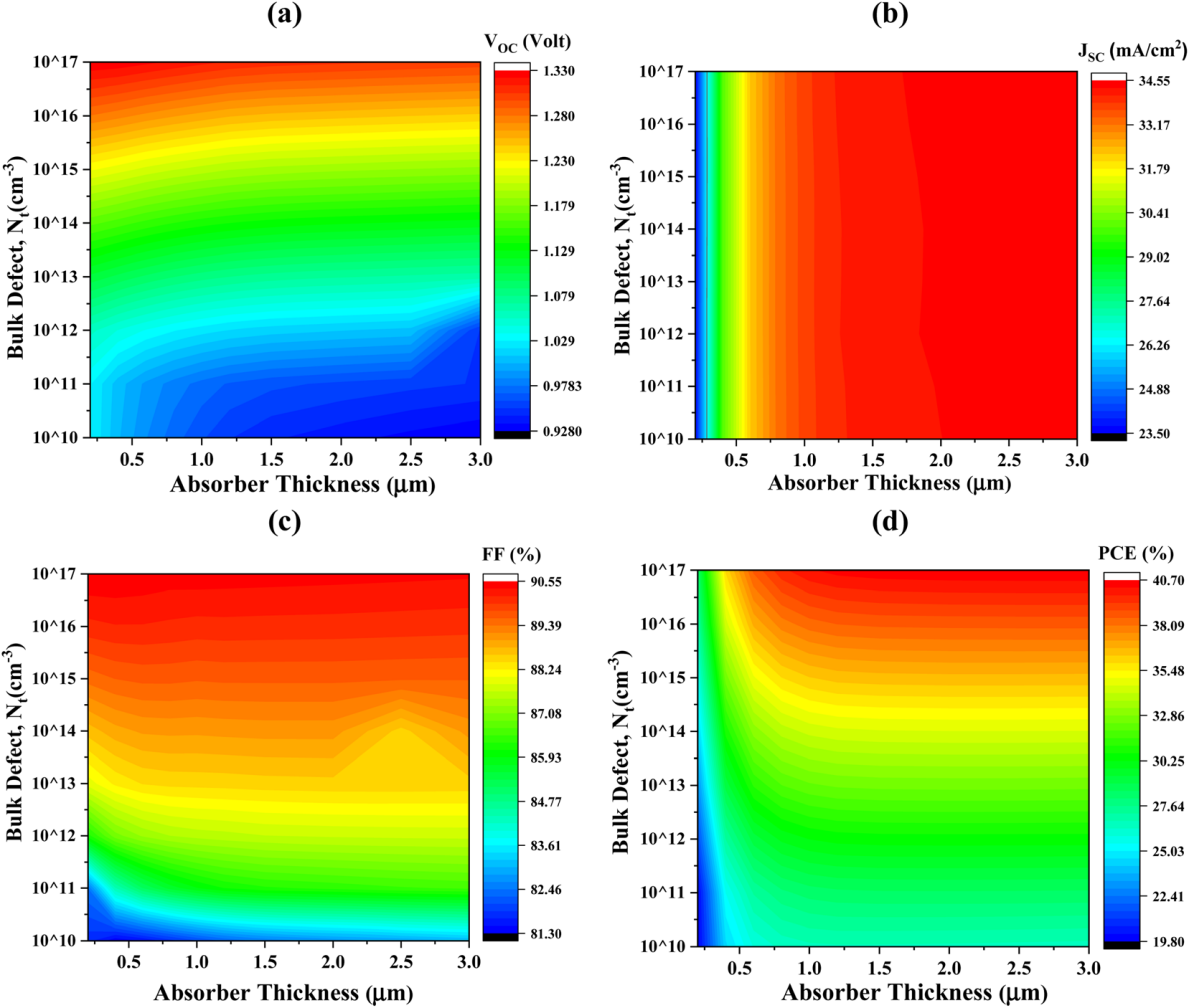
Concurrent effect of PV performance factors (a) *V*_oc_ (b) *J*_sc_ (c) FF and (d) PCE due to the alteration of absorber thickness and defect density of CIGS solar cell.

The impacts of altering the thickness and defect density of the CIGS absorber layer on the overall performance of the PV cell are shown in Fig. 4. Herein, the thickness and defect levels are varied in the ranges from 250 to 3000 nm and 10^10^ to 10^17^ cm^−3^, respectively. When the defect density of CIGS exceeds 10^12^ cm^−3^, the solar cell's efficiency begins to decrease abruptly. The PCE, FF, *J*_sc_, and *V*_oc_ of Al/FTO/SnS_2_/CIGS/Sb_2_S_3_/Ni structures decrease from 40.70 to 19.80%, 90.55 to 81.30%, 34.55 to 23.50 mA cm^−2^, and 1.33 to 0.92 V, when the defect density and the absorber layer thickness change from 250 to 3000 nm, and 10^10^ to 10^17^ cm^−3^, respectively.

The major change has been detected within ranges of thickness and defect density alteration on performance factors. The optimum *V*_oc_ of 1.08 V has been achieved for thicknesses 1000 nm and the defect density of 10^12^ cm^−3^, as illustrated in Fig. 4(a). Once the defect density exceeds the limit of 10^12^ cm^−3^, there is a prominent decrease in the *V*_oc_ to 0.74 V. It has been observed that *J*_sc_ reaches its highest value of 33.75 mA cm^−2^, at the thickness value of 1000 nm and defect densityof 10^12^ cm^−3^. If the defect's density is more than 10^13^ cm^−3^, a dramatic decline in *J*_sc_ has been demonstrated as the absorber's thickness decreases. The FF exhibits the same behaviors as *V*_oc_ represented in Fig. 4(c). In Fig. 4(d), it is demonstrated that the conversion efficiency attains its maximum value (>31%) in the range of 800–2000 nm thickness and exhibiting a defect density of up to 10^12^ cm^−3^. The amount of Shockley–Read–Hall (SRH) recombination increases because of the presence of defect in the CIGS absorber layer, which reduces the number of PGCs and reduces the values of *V*_oc_, *J*_sc_, FF, and PCE.^63^ The determine value of PCE 31.15%, with *V*_oc_ of 1.08 V, *J*_sc_ of 33.75 mA cm^−2^, and FF of 88.50%, has been attained using a optimized thickness of 1000 nm for the CIGS absorber layer and defect density of 10^12^ cm^−3^.

### 4.4 Effect of capacitance–voltage (*C*–*V*) on performances of CIGS solar cell

**Fig. 5:**
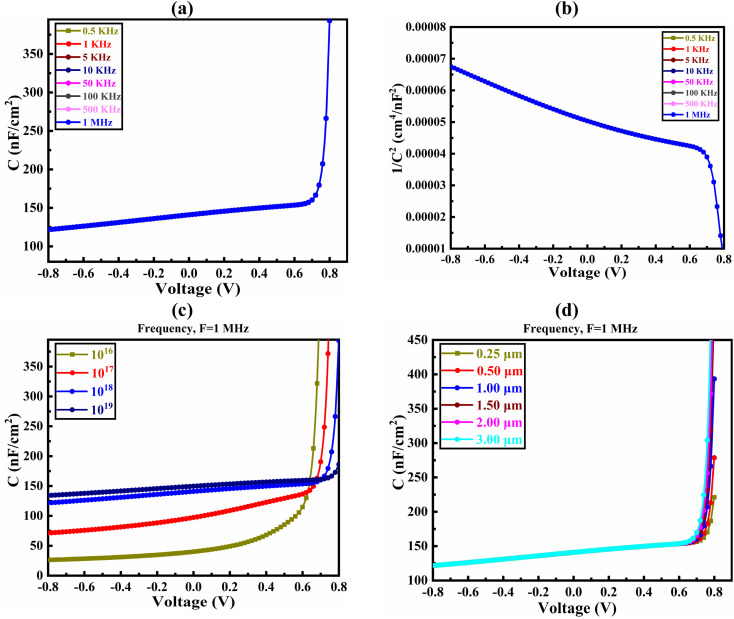
Effect of *C*–*V* on the CIGS solar cell observed through with the (a) frequency variation, (b) 1/*C*^2^–*V* curve, (c) absorber doping variation, and (d) absorber thickness variation.

The capacitance–voltage (*C*–*V*) investigation has been carried out in the frequency range varying from 0.5 kHz to 1 MHz to justify the coordination of the findings. The diffusion and depletion capacitances are associated with the p–n junctions. Diffusion capacitance predominates at forward bias voltage, meanwhile the depletion capacitance is higher in magnitude at reverse bias voltage. According to Fig. 5(a), a p–n junction PV cell's capacitance is 125 nF cm^−2^ under no bias voltage. The capacitance increases exponentially as the potential polarization at a specific frequency rises. The absorber traps are insensitive to frequency, which shows this tendency. Since the effective traps are inactive for reducing the effective charge at the reverse bias, the capacitance is decreased.^64^Fig. 5(b) illustrates the Mott–Schottky plot for the Al/FTO/SnS_2_/CIGS/Sb_2_S_3_/Ni heterostructure in the PV cell. The proposed SC's flat-band potential resulted from the voltage axis's junction with the 1/*C*^2^ plot. The p-type CIGS layer and the space-charge area are both primarily occupied by negative slope, which shows that holes are the majority of carriers.

The improved carrier density PGCs in the CIGS layer may be due to the exposure of sun light. The deviation in 1/*C*^2^ may exist due to the existence of localized significant levels in the absorber layer. The modulation of the bulk carriers has the paramount effect on the deep states.^65^ The *C*–*V* characteristics of CIGS SC are shown in Fig. 5(c) because of the alteration of doping concentration of the absorber layer. The capacitance values increase as the forward bias voltage increases, behaving like Mott–Schottky junctions. The Mott–Schottky plot reveals a low built-in potential value due to sunlight exposure, which is explicable by the capacitance created by photo-generated carriers in materials with lower mobility. The increase in doping density makes the accumulation of charges easier at the interface, which helps to increase the capacitance value that was reported in the previous study.^66^Fig. 5(d) illustrates the variation in capacitance at varying thicknesses with a constant frequency of 1 MHz due to changes in bias voltage. The change in capacitance due to thickness variation is virtually insignificant. After 0.80 V, the capacitance value increases sharply as the voltage rises. The similar type of capacitance tendency with respect to thickness and voltage has been reported in the earlier study.^67^

### 4.5 Influence of interface (SnS_2_ buffer/CIGS absorber) defect density

**Fig. 6:**
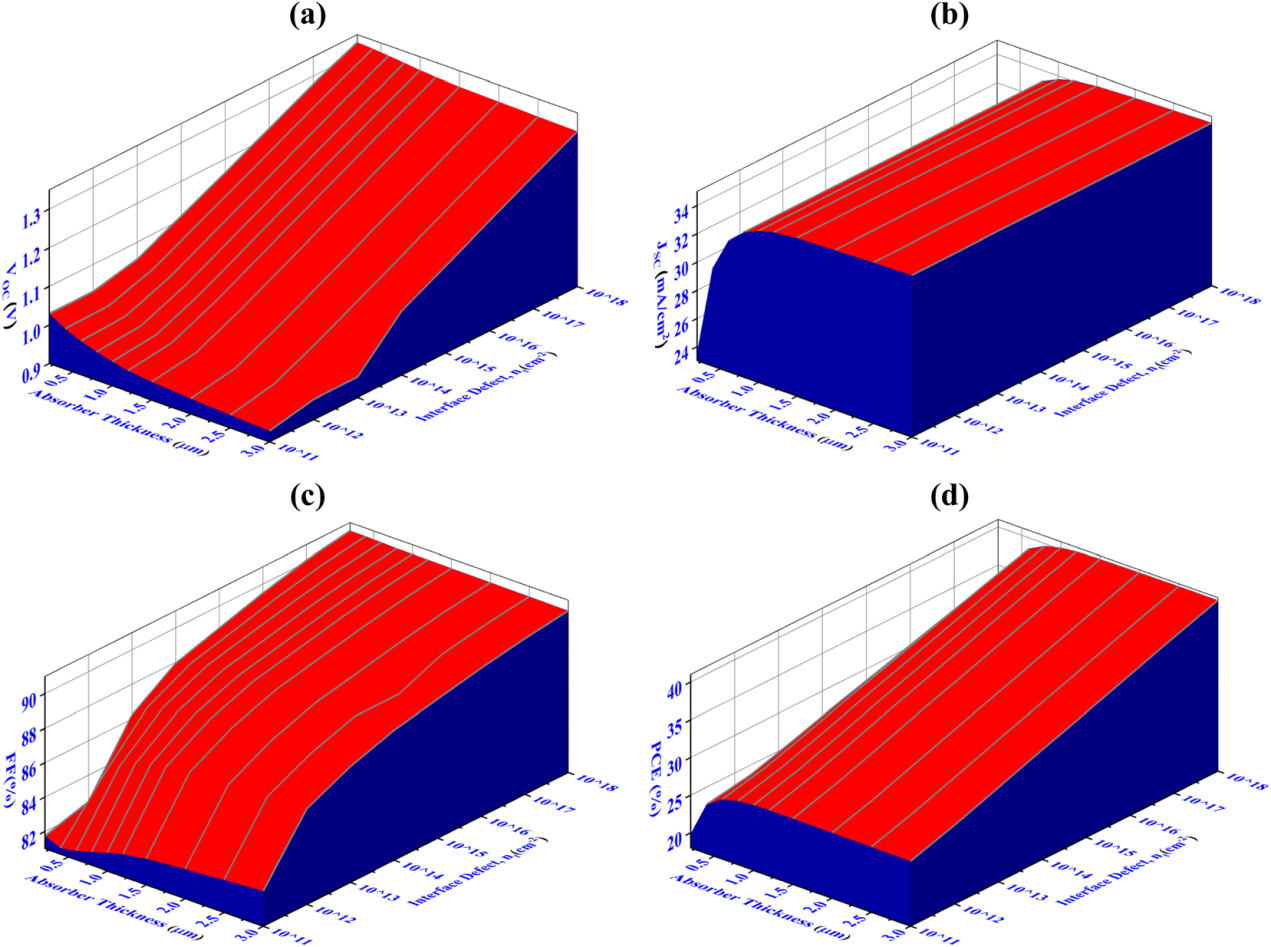
Concurrent impact of PV performance factors of (a) *V*_oc_ (b) *J*_sc_ (c) FF, and (d) PCE due to the variation of interface (SnS_2_ buffer/CIGS absorber) defect density.

Fig. 6 illustrates the impact of interface defects (*n*_t_) density between SnS_2_ buffer and CIGS absorber on the PV cell's performance. As the density of interface defects increases between the CIGS and SnS_2_ layers, the carriers recombination at the interface also enhances. Therefore, carriers are more expected to be traped in the interface, leading to low *J*_sc_ and decreased PCE.^68^ To explore the influence of defect density on the *J*–*V* properties, simulations has been conducted by modifying the *n*_t_ value across different active layers, ranging from 10^11^ cm^−2^ to 10^18^ cm^−2^. It has been observed from Fig. 6, that the value of PCE stays constant up to 10^12^, after that it starts to decrease. The SnS_2_/CIGS interface defect density has a less prominent impact on the *J*_sc_ and FF (%) as presented in Fig. 6. The optimum SnS_2_/CIGS interface defect density for the proposed design has been considered to be 10^12^ cm^−2^. This value is optimized considering for achieving high conversion efficiency, and the low cost manufacturing process.^54^

### 4.6 Effect of interface (CIGS absorber/Sb_2_S_3_ BSF) defect density

**Fig. 7:**
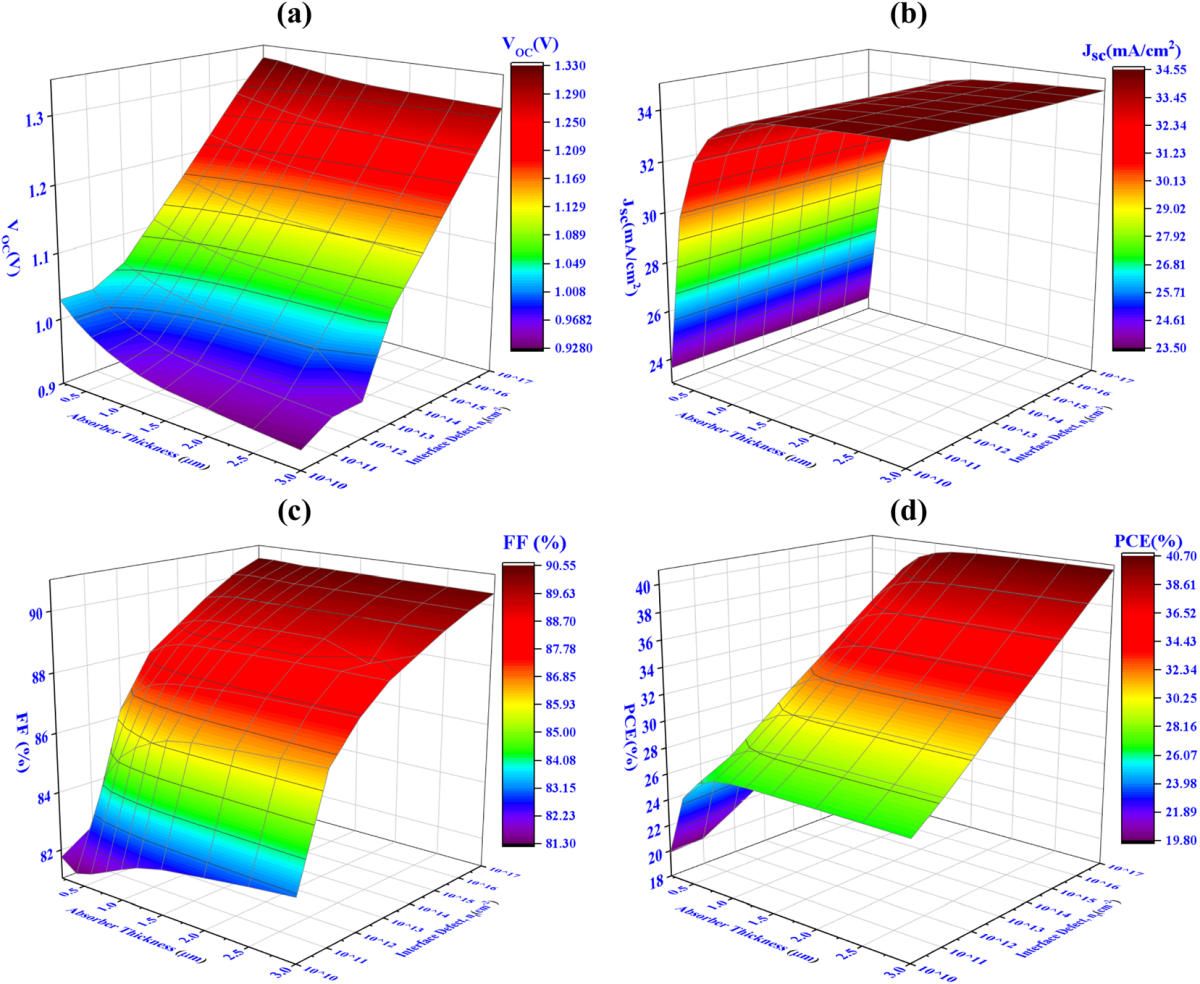
Concurrent effect of PV performance factors (a) *V*_oc_ (b) *J*_sc_ (c) FF, and (d) PCE due to the alteration of interface (CIGS absorber/Sb_2_S_3_ BSF) defect density.

The impact of interface defects density between the CIGS and Sb_2_S_3_ BSF layers on the solar cell's performance is shown in Fig. 7. Recombination of carriers at the interface between the CIGS and Sb_2_S_3_ BSF layers increases with the density of interface defects. Carriers are therefore more likely to become trapped in the interface, which lowers PCE and results in low *J*_sc_. By altering the defects density value across several active layers have been run to examine the impact of defects density on the *J*–*V* characteristics with range of 10^10^ cm^−2^ to 10^17^ cm^−2^. From figure, it can also be shown that the PCE increases steadily until 10^12^ cm^−2^, at which point it begins to decline. As shown in Fig. 7, the *J*_sc_ and FF (%) are less influenced by the CIGS/Sb_2_S_3_ interface defect density. After details investigation, the interface defect density of 10^12^ cm^−2^ is found at CIGS/Sb_2_S_3_ interface for the proposed solar cell structure. To attain optimal conversion efficiency and a low-cost production process, this value has been optimized.

### 4.7 Effect of temperature on performances of proposed CIGS solar cell with and without BSF layer

**Fig. 8:**
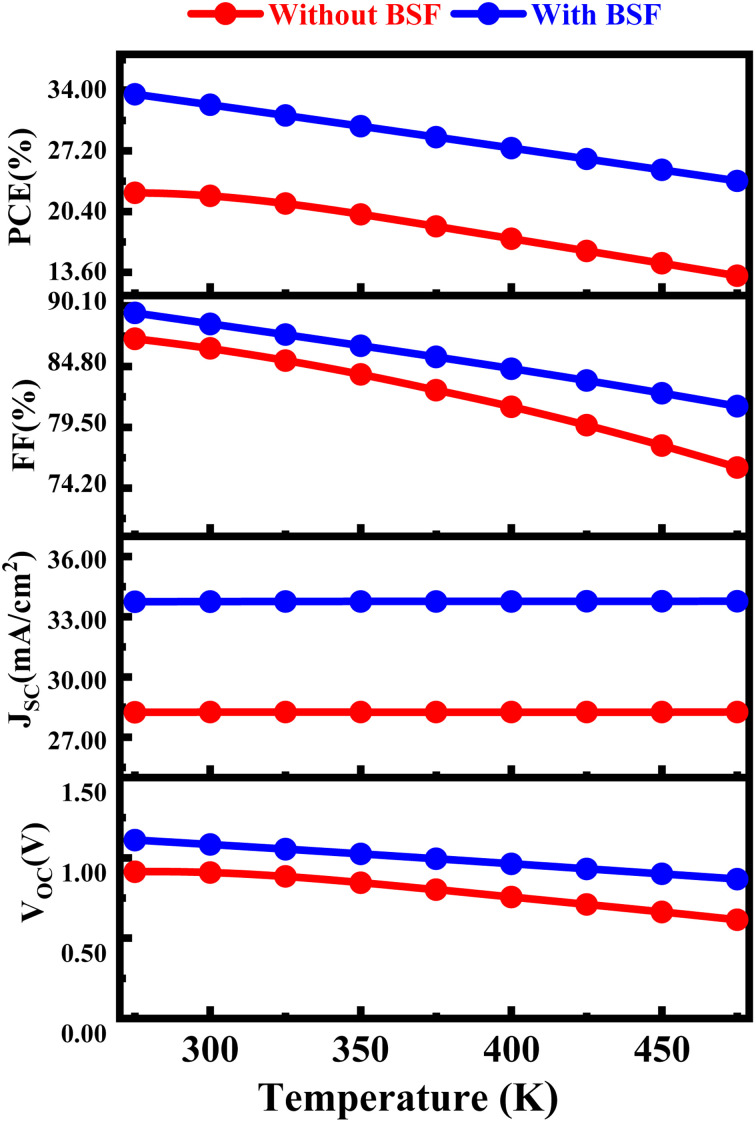
Influence of temperature on proposed CIGS solar cell with and without BSF layer.

The PV properties of CIGS-based solar cells with and without BSF undergo a significant reduction when the temperature is elevated from 275 K to 475 K is depicted in Fig. 8. The PCE of the FTO/SnS_2_/CIGS/Sb_2_S_3_ and FTO/SnS_2_/CIGS structures are observed to be 31.15% and 22.14%, respectively, at a temperature of 300 K. As the temperature of CIGS-based solar cells with and without BSF surges from 275 K to 475 K, the PV properties experience a significant decline. For instance, the PCE values, which are initially impressive, deteriorate to 25.4% and 14.18%, respectively, at the above mentioned temperature. The outcomes of the simulations indicate that CIGS-based solar cells with BSF exhibit higher resilience to thermal stress than those without BSF.^31^ With a constant value of *J*_sc_, higher temperatures and fixed irradiance lead to the production of more electron–hole pairs. The effective energy gap of CIGS decreases with a rise in operating temperature, leading to an elevation in the reverse saturation current, which contributes to reduces *V*_oc_, FF, and efficiency values. The operating temperature of a PV solar cell affects its bandgap energy, which decreases as the temperature increases. Consequently, there is a slight rise in *J*_sc_ but a decline in *V*_oc_. Because of the reduction in *V*_oc_ and a marginal rise in *J*_sc_, the FF and PCE of the solar cells decrease at elevated temperatures.^69^

### 4.8 Current density voltage (*J*–*V*) and quantum efficiency (QE) properties of proposed CIGS solar cells

**Fig. 9:**
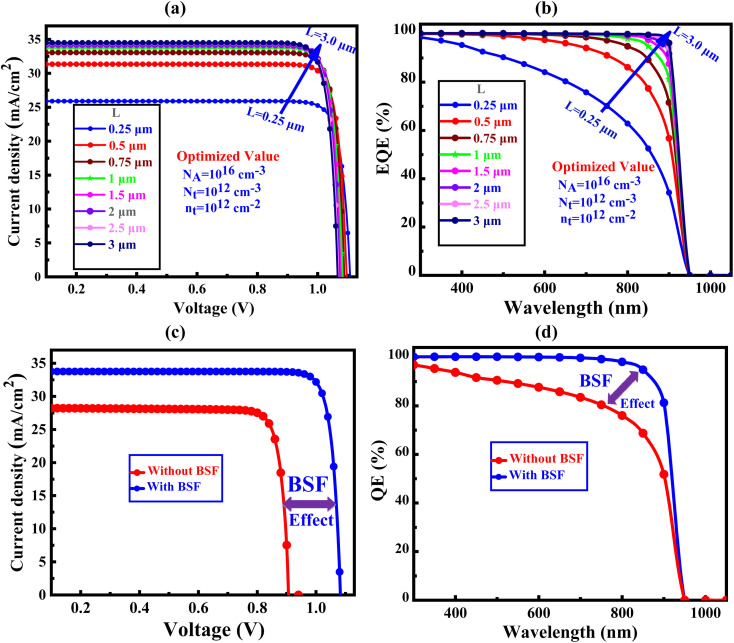
Impact of absorber layer thickness with Sb_2_S_3_ BSF layer on the (a) *J*–*V*, and (b) QE, respectively and optimized value of proposed structure of (c) *J*–*V* characteristics, and (d) QE curve comparison between with and without BSF.

The *J*–*V* and QE characteristics curves of proposed solar cells have shown in Fig. 9. In Fig. 9(a) and (b) have shown the *J*–*V* and QE characteristics curves of the proposed solar cells with various absorber thicknesses with BSF, respectively. The insertion of BSF ehnaces the *J*–*V* and QE at lower thickness of CIGS absorber layer as indicated in Fig. 9(c) and (d). After details investigation, the optimal PCE value of 22.14% with *V*_oc_ of 0.91 V, *J*_sc_ of 28.21 mA cm^−2^, and FF of 86.31 is found without BSF. On the other hand, using Sb_2_S_3_ BSF in the structure (FTO/SnS_2_/CIGS/Sb_2_S_3_/Ni), the PCE is improved to 31.15% with *V*_oc_ of 1.08 V, *J*_sc_ of 33.75 mA cm^−2^, and FF of 88.50, respectively.^70,71^ In addition, we find a small variation in *V*_oc_ for the absence of BSF but a amazing impact of *V*_oc_ is found with BSF (Sb_2_S_3_). Actually, Fig. 9(c) and (d) depict the optimized *J*–*V* curve and the equivalent QE spectrum for the SC with and without BSF layer.

The *J*–*V* curve for the CIGS-based SC device with and without the BSF (Sb_2_S_3_) layer is shown in Fig. 9(c) following the optimization of parameters including carrier transport layers (CTLs) thickness, defect density, temperature, interface defect density, and resistances.^72,73^ The inclusion of the BSF layer enhances the QE as represented in Fig. 9(d), this leads to an increase in the *J*_sc_ due to the reduction of the rate of the carrier recombination at the back electrode for existence of the back electric field. In this situation, the *J*_sc_ value reaches its maximum of 33.75 mA cm^−2^. The energy gap of the absorber material determines the extent of current improvement.^74^ By elevating the natural potential at the absorber interface, the *V*_oc_ value is enhanced, to achieve a substantial built-in potential indicating that pave the path for obtaining high-performance solar cells.

## 5 Comparison between related published work and proposed work for CIGS cells with and without BSF

A comparison of the related published work and the proposed work, with and without the inclusion of BSF, is presented in Tables 3 and 4, respectively. The superiority of the suggested cell structure FTO/SnS_2_/CIGS/Sb_2_S_3_/Ni over other cell structures is attributed to its remarkable performance in terms of high *J*_sc_ and *V*_oc_, leading to increased PCE. CIGS can absorb a large amount of sunlight with a comparatively thin layer of material due to high absorption coefficient. This makes it possible to produce TFSCs, which require less material to produce while still absorbing a sizable amount of sunlight. On the other hand, by changing the proportions of copper, indium, gallium, and selenium, the bandgap of CIGS can be tuned. Because of its tunability, the solar cell's performance may be adjusted to more closely resemble the sun spectrum, which boosts efficiency all around then other absorber.^75^ The insertion of BSF between CIGS active layer and the back electrode contribute to create an electric field which in turn inhibit carrier recombination of electron hole pair, therefore *J*_sc_ and *V*_oc_ enhances as well as the PCE also increases. If the manufacturing process of the proposed cell structure can be executed successfully, this design approach will emerge as the optimal choice.

**Table 3:** PV performance of suggested cell compared to other reported CIGS solar cell without BSF

Types of research	CIGS layer thickness (μm)	*V*_oc_ (V)	*J*_sc_ (mA cm^−2^)	FF (%)	*η* (%)	Ref.
Experimental	2.0	0.671	34.90	77.60	18.10	76
Experimental	1.0	0.689	35.71	78.12	19.20	77
Experimental	2.2	0.690	35.50	81.20	19.90	78
Experimental	—	0.741	37.80	80.60	22.60	79
Theoretical	1.0	0.743	34.47	83.09	21.30	13
**Theoretical**	**1.0**	**0.91**	**28.21**	**86.31**	**22.14 (without BSF)**	**This work**

**Table 4:** Impact of BSF layer in comparison with related research

Types of research	Absorber	BSF	*η* without BSF (%)	*η* with BSF (%)	Ref.
Experimental	Si	ZnS	6.40	11.02	80
Experimental	Si	Al	12.96	13.75	81
Experimental	CIGS	MoSe_2_	9	14	82
Theoretical	CdTe	V_2_O_5_	19.58	23.50	83
Theoretical	CZTS	CZTS	12.05	14.11	84
Theoretical	ZnTe	Sb_2_Te_3_	7.14	18.33	85
Theoretical	CZTSSe	SnS	12.30	17.25	86
Theoretical	CIGS	Si	16.39	21.30	13
Theoretical	CIGS	μc-Si:H	19.80	23.42	87
Theoretical	CIGS	SnS	17.99	25.29	88
Theoretical	CIGS	PbS	22.67	24.22	89
**Theoretical**	**CIGS**	**Sb**_**2**_ **S**_**3**_	22.14*	**31.15**	**This work**

## 6 Conclusions

This paper presents the amazing performance of double heterojunction (DH) solar cells based on CIGS absorber. In this study, a novel solar cell structure of FTO/SnS_2_/CIGS/Sb_2_S_3_/Ni is investigated utilizing the SCAPS-1D simulation software to increase all PV performance characteristics. The thickness, acceptor density, defect density, capacitance–voltage (*C*–*V*), interface defect density, rates of generation and recombination, operating temperature, current density, and quantum efficiency of the absorber, buffer, and BSF layer have all been studied with and without BSF. The device's performances, including FF, PCE, *J*_sc_, and *V*_oc_ are significantly impacted by the existence of the BSF layer. After details optimization the optimal thicknesses for the FTO window, CIGS absorber, SnS_2_ buffer, and Sb_2_S_3_ BSF layers are found to be 0.05 μm, 1.0 μm, 0.05 μm and 0.20 μm, respectively. The large thickness of buffer creates the series resistance and absorption losses in the solar cell structure. Since the buffer allows light to enter the solar cell device, so excellent transparency and the right thickness are needed, which is the 0.05 μm for SnS_2_ buffer in our proposed structure. Finally, this proposed novel DH solar cell structures exhibit an efficiency of 31.15% including *V*_oc_ of 1.08 V, *J*_sc_ of 33.75 mA cm^−2^, and FF of 88.50%. The outcomes of this study provide insights into the development of an ultra-thin Sb_2_S_3_ BSF layer, which can be included in conventional CIGS solar cells to improve their efficiency and reduce the absorber material's cost.

## Abbreviations

CIGS: Copper indium gallium selenide
Sb_2_S_3_: Antimony trisulfide
SnS_2_: Tin(iv) sulfide
FTO: Fluorine-doped tin oxide
BSF: Back surface field
*V*_oc_
: Open circuit voltage
*J*_sc_
: Short circuit current
FF: Fill factor
PCE: Power conversion efficiency
*C*–*V*: Capacitance–voltage
SRV: Surface recombination velocity
TMDs: Transition metal dichalcogenides
CBM: Conduction band minimum
CBO: Conduction band offset
TW: Terawatts
TFSC: Thin film solar cell
SC: Solar cell
PV: Photovoltaic
CZTS: Copper zinc tin sulfide
HTL: Hole transport layer
PGC: Photo generated carrier
PGHs: Photo generated holes
PGEs: Photo generated electrons
EQE: External quantum efficiency
SRH: Shockley read hall
SD: Single-donor
PDT: Post-deposition treatment
VB: Valence band

## Ethical approval

The all authors declare that the manuscript does not have studies on human subjects, human data or tissue, or animals.

## Author contributions

Md. Ferdous Rahman: conceptualization, methodology, software, validation, formal analysis, visualization, investigation, data curation, supervision, writing – original draft, review & editing. Mithun Chowdhury: methodology, software, validation, formal analysis, visualization investigation, data curation, writing – original draft, review & editing, Latha Marasamy, Mustafa K. A. Mohammed, Md. Dulal Haque, Sheikh Rashel Al Ahmed, Ahmad Irfan, Aijaz Rasool Chaudhry, Souraya Goumri-Said: validation, formal analysis, writing – original draft, review & editing.

## Conflicts of interest

The authors have no conflicts of interest.
